# The Interrelationship between Promoter Strength, Gene Expression, and Growth Rate

**DOI:** 10.1371/journal.pone.0109105

**Published:** 2014-10-06

**Authors:** Matthew S. Bienick, Katherine W. Young, Justin R. Klesmith, Emily E. Detwiler, Kyle J. Tomek, Timothy A. Whitehead

**Affiliations:** 1 Department of Chemical Engineering and Materials Science, Michigan State University, East Lansing, Michigan, United States of America; 2 High School Honors Science/Mathematics/Engineering Program, Michigan State University, East Lansing, Michigan, United States of America; 3 Department of Biochemistry and Molecular Biology, Michigan State University, East Lansing, Michigan, United States of America; 4 Department of Biosystems and Agricultural Engineering, Michigan State University, East Lansing, Michigan, United States of America; Virginia Commonwealth University, United States of America

## Abstract

In exponentially growing bacteria, expression of heterologous protein impedes cellular growth rates. Quantitative understanding of the relationship between expression and growth rate will advance our ability to forward engineer bacteria, important for metabolic engineering and synthetic biology applications. Recently, a work described a scaling model based on optimal allocation of ribosomes for protein translation. This model quantitatively predicts a linear relationship between microbial growth rate and heterologous protein expression with no free parameters. With the aim of validating this model, we have rigorously quantified the fitness cost of gene expression by using a library of synthetic constitutive promoters to drive expression of two separate proteins (eGFP and amiE) in *E. coli* in different strains and growth media. In all cases, we demonstrate that the fitness cost is consistent with the previous findings. We expand upon the previous theory by introducing a simple promoter activity model to quantitatively predict how basal promoter strength relates to growth rate and protein expression. We then estimate the amount of protein expression needed to support high flux through a heterologous metabolic pathway and predict the sizable fitness cost associated with enzyme production. This work has broad implications across applied biological sciences because it allows for prediction of the interplay between promoter strength, protein expression, and the resulting cost to microbial growth rates.

## Introduction

Quantitative understanding of the fitness cost of gene expression is important for fields as diverse as synthetic biology, metabolic engineering, evolutionary biology, and applied microbial physiology. In this work, the fitness cost for a given strain is defined as the reduction in growth rate upon a specific gene expression relative to no gene expression. It has been understood for at least a generation that heterologous protein expression exerts a fitness cost on the host organism [1], [2] with approximately a linear relationship between gene expression and growth rate [3]–[6]. The mathematical models proposed in some of these previous studies sufficiently corroborated cellular component mass balances and fitted empirical relationships to experimental data, though the form of these early models yielded little predictive value. Ideally, a model with few or zero free parameters that allows quantitative prediction of fitness cost *a priori* would enhance a model's utility for forward engineering of microorganisms.

Recently, Scott et al. revisited existing empirical relationships relating specific growth rates to RNA/protein ratios [7]. From these results, a growth theory model was proposed stating that growth rates are limited by mRNA translation of a proteome fraction apportioned to match the nutrient influx, along with a fraction of ribosome-affiliated proteins needed for protein synthesis. According to this model, increased expression of unnecessary heterologous protein will decrease the proteome fraction allocated for synthesis of ribosome associated proteins and hence the growth rate. This model predicts that for expression of every 1% of heterologous protein per dry cell weight, the relative growth rate is reduced by ∼3% through a single non-dimensional equation containing no free parameters. Validation of this model would have profound implications on our ability to forward engineer biological systems, albeit with some known limitations [8]–[10]. As one example, metabolic engineers often need to express high amounts of heterologous enzymes to support flux through a given pathway; this overexpression is often described as a “metabolic load” or a “metabolic burden” [11]–[13]. Precise quantification of this metabolic load would significantly reduce experimental search space in flux optimization.

However, the prediction of the fitness cost of gene expression has not been rigorously assessed beyond a handful of systems. Scott et al. [7] used IPTG to induce a lac promoter driving **β**-galactosidase expression in *E. coli* in three different growth conditions and confirmed the fitness cost of gene expression to be consistent with their ribosome allocation model. However, IPTG induction is known to result in bimodal gene expression in a wide range of conditions, potentially clouding results by introducing cell heterogeneity [14]. Assessing the fitness costs of unimodal gene expression driven by different constitutive promoter strengths provides an alternative way to rigorously test of the ribosome allocation model. Testing on constitutive promoters will also serve another purpose: with the advent of synthetic promoters in a range of model organisms, flux optimization in metabolic engineering often occurs by transcriptional engineering [15], [16]. Activities of synthetic promoters are often reported in relative terms [17], and for constitutive promoters overall steady state protein concentrations are known to scale inversely with cellular growth rates [18]. Incorporating ribosome allocation model with known relationships between promoter activity and growth rates would enable quantitative prediction of the interrelationship between promoter strength, gene expression, and growth rates.

With the aim of validating these proposed relationships, we generated a library of synthetic promoters, which were used to drive expression of two separate proteins under different media conditions and strains. We find that the ribosome allocation model sufficiently explains the fitness cost of gene expression. Furthermore, we find that a model based on basal promoter strength can be used to predict gene expression and fitness costs across different growth media in *E. coli*. Combined, these results suggest a surprising simplicity to the interrelationships between promoter strength, gene expression, and exponential growth rate in bacteria.

## Materials and Methods

### Reagents

All chemicals were purchased from Sigma-Aldrich (St. Louis MO, USA), except where noted. All primers were purchased from IDT (Coralville IA, USA). Sequences of all genetic constructs used in this study were verified by Genewiz (South Plainfield, New Jersey) and are listed in **Note S1**. Representative constructs have been made available on the AddGene plasmid repository (www.addgene.org).

### Preparation of plasmids

The starting plasmid pJK_proB_eGFP was created by modifying the promoter and antibiotic resistance gene of pET-29b(+) (Novagen). The proB promoter sequence [19] was ordered as a gBlock (IDT) and cloned into pET-29b(+) between the BglI and XbaI restriction sites using standard techniques. On pET-29b(+) the lacI, lacO, and the T7 promoter were removed between these restriction sites. The antibiotic resistance gene on pET-29b(+) was swapped to TEM-1 BLA (Amp_R_) from pET-22b(+) (Novagen) using Gibson assembly [20]. eGFP (BBa_E0040) from the BioBrick collection (partsregistry.org) was cloned in-frame between the NdeI and XhoI restriction sites using Gibson assembly. The full-length construct includes the eGFP sequence with a C-terminal LEHHHHHH sequence (27.98 kDa expected MW). The ribosome binding site (sequence AGGAG), pMB1 ori, and the T7 terminator were not modified during the creation of the base plasmid (full sequence listed in **Note S1**).

Five promoter libraries were created in the −35 (NNTACG, TTNNCG, TTTANN) and −10 (NNATAT, TANNAT) regions of the proB promoter. Mutagenic primers were designed using QuikChange Primer Design software (Agilent) and libraries created by Kunkel mutagenesis [21]. Libraries were individually transformed into *E. coli* TUNER [F– ompT hsdSB (r_B_– m_B_–) gal dcm lacY1] (Novagen). From each plate, thirty-six colonies were chosen spanning the range of colony fluorescence, grown, flash frozen, and stored at −80°C. 22 representative sequences were selected for further testing (**Table S1**).

To prepare the amidase constructs, a codon optimized gene *amiE* encoding an aliphatic amidase from *Pseudomonas aeruginosa* [22] was custom ordered from Genscript with flanking NdeI/XhoI restriction sites, and cloned in-frame to the appropriate pJK-series or pET29 (Novagen) plasmid using standard methods. The full-length protein has a predicted MW of 39.0 kDa. Plasmids were transformed into *E. coli* TUNER and *E. coli* MG1655rph+ [F^−^ λ^−^] (*E. coli* genetic stock center, Yale University, New Haven, CT). Strains were stored as above. Tested sequences are listed in **Table S2**.

### Growth Conditions and Determination of Growth Rates

*E. coli* strains were grown in one of three media conditions with carbenicillin: M9 salts plus 4 g/L glucose (M9), M9 salts plus 0.2%(w:v) casamino acids plus 4 g/L glucose (M9-CA), or LB [23]. Cells were taken from −80°C freezer stocks and incubated at 37°C and 900 rpm overnight in a Heildoph Titramax 1000 plate shaker (Heildoph Instruments). The next morning, 37°C pre-warmed Hungate tubes (125 mm height, 14 mm inner diameter; Chemglass Life Sciences) containing 4.0 mL of appropriate media were inoculated with cells from overnight culture at a starting OD_600_ of 0.02. The tubes were incubated at 37°C and shaken at 250 rpm in New Brunswick I-26 shaker with a 30° tilt angle for the test tube holder. OD_600_ measurements using a Genesys 20 spectrophotometer (Thermo Scientific) were taken until the culture OD_600_ approached 0.6, and were taken at least two independent times.

### Flow cytometry

Cells were sampled at exponential growth (0.15≤OD_600_≤0.3) and diluted 50-fold into phosphate buffered saline. Cells were immediately processed on an Accuri C6 Flow Cytometer (BD), and eGFP fluorescence was recorded on the FL-1 channel using a 510±7.5 nm filter.

### Determination of conversion factor between OD_600_ and dry cell weight

Cells were inoculated in 50 mL of one of the three media conditions in a 250 mL flask and incubated overnight at 37°C and 250 rpm in a New Brunswick I-26 shaker. The next morning, 500 mL of pre-warmed fresh media was inoculated to a starting OD_600_ of 0.02 and incubated at 37°C and 250 rpm. When cultures reached an OD_600_ of 0.4 and, separately, 0.7, 200 mL of the culture was transferred to a pre-chilled 500 mL container and placed in an ice bath for 10 minutes. Cells were pelleted at 4°C and 8,000 rpm and resuspended in nanopure water three times. The final centrifugation step was done in a pre-weighed polypropylene Falcon tube (Fisher), which was then fitted with qualitative cellulose filter paper (Whatman) and placed in a 50°C isotemp oven (Fisher Scientific) for 48 hr. Controls were done to correct for Falcon tube mass lost during the drying step. For each media condition this experiment was performed at least two independent times. The conversion factors are listed in **Table S3** and are consistent with other experimental determinations of optical density conversions to dry cell weight [24]. There was little difference in the conversion factors between the two strains tested, the three different media conditions, and between strains expressing high or low amounts of heterologous protein.

### eGFP quantification

200 **µ**L of culture was sampled at exponential growth (0.15≤OD_600_≤0.3), and the fluorescence (RFU_GFP_) immediately measured using a Synergy H1 Hybrid Microplate Reader (Biotek) (excitation wavelength 481 nm, emission wavelength 507 nm, gain 50, and read height 7 mm) in 96-well black microtiter plates. By comparing cell fluorescence to a standard curve, sample eGFP concentration (**Φ**_u_) was determined according to the following equation:
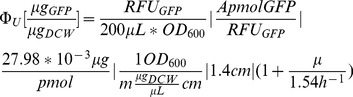
(1)


Where A is the conversion between pmol GFP and the standard curve, m is the conversion between dry cell weight and OD_600_ determined for different growth media in this study, 1.4 cm is the pathlength through the Hungate tubes, and the right-hand term corrects for GFP maturation time [25]. For each strain, eGFP was quantified at least two independent times.

To prepare the eGFP standard, the plasmid pJK_proB_eGFP was transformed into *E. coli* TUNER. The next day, a single colony was picked and inoculated into 400 mL LB with 400 µL carbenicillin. The flask was incubated at 37°C and shaken at 250 rpm overnight. The next morning, the cells were pelleted at 4000 rpm for 15 minutes, resuspended in 20 mL resuspension buffer (50 mM Tris-HCl pH 8.0, 50 mM NaCl, and 15 mM imidazole), and pelleted again at 4000 rpm for 10 minutes. Resuspension buffer was added to cells at a ratio of 3 mL buffer:1 g wet cell weight, along with 12 **µ**L DNAse, 12 **µ**L lysozyme, and 10 **µ**L PMSF. The cell suspension was sonicated, incubated at 30°C for 15 minutes, and then centrifuged at 15000 g for 20 minutes at 4°C. The supernatant was applied to a Ni-NTA agarose affinity column (Qiagen), and eGFP was eluted with 2.5 mL of buffer containing 50 mM Tris-HCl (pH 8.0) and 400 mM imidazole. eGFP was desalted using gravity flow PD-10 desalting columns (GE Healthcare) into 3.5 mL phosphate buffered saline, pH 7.5. The protein purity was at least 95% as determined by SDS-PAGE. The concentration of the eGFP standard was determined by absorption intensity of eGFP at 448 nm by the NaOH denaturation method [26] using the published extinction coefficient of 44,100 M^−1^cm^−1^.

### Amidase quantification

1000 **µ**L of culture was sampled at exponential growth (0.15≤OD_600_≤0.3), pelleted immediately at 10,000×g for 5 min, and resuspended in PBS. OD_600_ was recorded using a cuvette with a 1 cm pathlength, and cells lysed by sonication using a 120 W, 20 kHz FB120 sonicator (Fisher Scientific) with a 1/8″ sonicator horn using the settings: 39 s total on time, cycled for 3 s on, 15 s off, 37% amplitude. Controls were done to ensure that ≥95% of total activity was recovered during this initial lysis step. The supernatant was clarified by centrifugation at 15,000×g for 5 min, and amiE activity quantified by determination of free ammonia by a phenol nitroprusside microplate method [27]. Briefly, supernatant from each lysate was diluted into PBS and incubated at room temperature with 50 mM filter-sterilized acetamide at a total volume of 1 mL. Every 3–5 minutes, 100 **µ**L of sample was drawn and reaction stopped by pipetting into a microwell plate with 50 **µ**L ice-cold phenol nitroprusside. At the end of the last time point, 50 **µ**L of alkaline hypochlorite solution was added to each well. The plate was incubated for 40 min at 35°C in a Synergy H1 Hybrid Microplate Reader (Biotek). A_625_ was continuously monitored during the incubation, and endpoint absorbance measurements were taken once stabilized. By comparing sample velocities to velocities of known amounts of amiE standard, the soluble amidase concentration (**Φ**_u_) for each sample was determined according to the following equation:
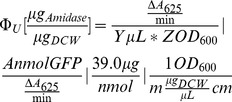
(2)


Where Y is the amount of lysate in the 1 mL reaction volume, Z is the sample OD_600_ at the time of lysis, A is the conversion between nmol amidase and the reaction velocity as determined from the standard curve, and m is the conversion between dry cell weight and OD_600_ determined for different strains. Representative sample data of the assay endpoint data is shown in **Figure S3**. Tested sequences are listed in **Table S2**. For each strain, amiE was quantified at least three independent times.

The amidase standard was prepared by transforming pET29-amiE into *E. coli* BL21* (DE3) and producing protein by auto-induction [28]. Protein was purified using the same method as eGFP except that a Talon metal affinity column (Clontech) was used and the protein was desalted into PBS, quantified by A_280_ using the predicted theoretical extinction coefficient of **ε**_280_ = 56,980 M^−1^cm^−1^, diluted to 60 **µ**M in PBS, and stored at 4°C until use. Controls were done to ensure that protein activity stored at 4°C remained constant for the duration of the experiment.

Western blots of amiE were performed on soluble, insoluble, and total cell extracts of *E. coli* strains harboring plasmids pJK_proB_amiE and pJK_pro6_amiE. Briefly, the soluble and insoluble portions of 0.5 or 1 **µ**g total cell lysate was run on 10–20% Tris-HEPES (Thermo scientific) denaturing gel electrophoresis and then transferred to nitrocellulose filter paper using an iBlot (Invitrogen). amiE was probed using an Anti-6X His tag Antibody (HRP) (abcam, catalog # ab1187) and visualized using enhanced DAB substrate (Thermo scientific) according to standard protocols. Gel densitometry was quantified using ImageJ [29]. All data was collected in triplicate on separate days. Total amidase concentration was found by summing the soluble and insoluble concentrations.

### Beta lactamase quantification

Cell lysates were prepared as above for the amiE quantification except that lysates were prepared in 50 mM sodium phosphate buffer, pH 7.0. TEM-1 beta lactamase (BLA) activity was quantified by incubating cell lysates with 62.5 **µ**M nitrocefin in 50 mM sodium phosphate buffer, pH 7.0 at 30°C and monitoring A_482_ for 45 min. Velocity was converted to mass of BLA relative to total dry cell weight using known catalytic parameters of TEM-1 BLA on nitrocefin under the assay conditions [30].

## Theory

### Ribosome allocation model

The general ribosome allocation model of gene expression cost has been laid out in previous work [7], [10]. Only the salient details are reported here. Consider a four-component proteome consisting of unneeded heterologous protein (**Φ**_U_), ribosomal-associated proteins (**Φ**_R_), non ribosomal-associated proteins that are growth-rate dependent (**Φ**_P_), and the remaining proteins (**Φ**_Q_). Variables for these proteins are normalized to total protein. By definition:
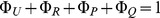
(3)


Two of these terms can be related to cellular growth rate as previously shown from empirical correlation [7]:
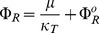
(4)

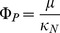
(5)


Where **μ** is specific growth rate, K_T_ and K_N_ are scaling constants, 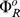 is an intercept under conditions of no growth. Empirical relation (4) above states that the ribosomal associated proteins (and, thus, active ribosomes) are linearly proportional to the exponential growth rate, and relation (5) sets a fraction of the proteome that is non-ribosome associated to be linearly proportional to the growth rate. These empirical relations, combined with the mass balance of the proteome, serve as the basis for the ribosome allocation model. Substituting in terms and solving for the dependence of growth rate, **μ**, on unnecessary protein:
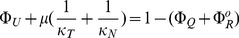
(6)


Defining **μ**_max_ as the maximum growth rate of the strain that can be supported in a given media in the absence of unneeded protein expression:
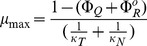
(7)


Which leads to the main equation tested in the present work:
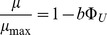
(8)


Φ_U_ is now expressed in more convenient units of protein mass per dry cell weight, and the constant b is defined as:
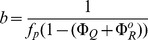
(9)


Where f_P_ is the fraction of dry cell weight that is protein. Previous experimental results [7], [31] set a lower and upper bound for the parameter b in *E. coli* as:
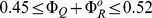
(10)

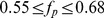
(11)

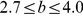
(12)


Thus, this ribosome allocation model predicts bacterial growth rate dependence to be linearly dependent on unnecessary protein production with zero free and two fixed parameters – parameter b, which is set between 2.7–4.0 as shown above, and μ_max_, which is the growth rate of the strain in the media condition in the absence of unnecessary protein expression. This latter parameter can be independently measured.

### Gene expression model

Consider the case of constitutive expression of an unneeded protein (Φ_U_) from cellular mRNA (R). Mass balances on the unneeded protein (Φ_U_) and cellular mRNA ([R]) entail:
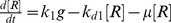
(13)

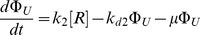
(14)


Where k_1_ is the transcription rate in units of mol mRNA per gene copy per time, g is the gene number per unit mass, k_d1_ is the degradation rate of mRNA, k_2_ is an effective translation rate of the unneeded protein in units of mass protein per mole mRNA per time, and k_d2_ is the degradation rate of the protein. Growth rates are included on the right hand side of these balances for concentration dilution by cell growth. Assuming steady state conditions during exponential growth, the fraction of unneeded protein can be expressed as:
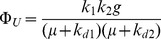
(15)


Making the simplifying assumption that growth rate is much slower than the mRNA degradation rate (for bacteria, typically on the order of seconds to minutes) and much faster than the protein degradation rate (on the order of tens of hours):
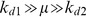
(16)


Leads to the following expression:
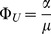
(17)

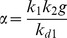
(18)


Making a further simplifying assumption that α does not depend on growth rate, and substituting (17) into (8) leads to the following promoter activity relationship:
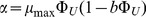
(19)


Using a limited dataset, Hintsche and Klumpp [32] recently proposed that the promoter activity scales with growth rate according to:
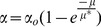
(20)where α_o_ is the portion of the bundled promoter activity that is invariant with respect to growth rate and μ* is a fitted parameter that describes the growth dependence on promoter activity. We can rewrite α_o_ solely in terms of expressed protein fraction by substituting (8) into (20):
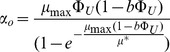
(21)


This combined promoter activity/growth rate model has a single adjustable parameter, μ*. Hintesche and Klumpp use a fitted value for μ* = 0.33 h^−1^, whereas we found that μ* = 0.40 h^−1^ fit our eGFP datasets marginally better. The molecular underpinnings of this exponential decay term is unclear and could reflect decreased mRNA stability, decreased basal transcription rate by RNA polymerase, decrease in plasmid copy number per unit mass, decreases in protein translation rates and/or efficiencies, or a combination of some or all of these terms. A more fundamental understanding of this exponential decay term is clearly needed. Regardless, combining the gene expression and ribosome allocation models allows quantitative determination of the dependence of growth rate and gene expression on promoter strength with a single fitted parameter.

### Explicit assumptions and limitations in the model

Owing to the simplicity of the model, there are explicit assumptions beyond the ones described above. These assumptions, and conditions under which they are expected to fail, include:

- These derivations only apply under conditions of steady state, balanced exponential growth. Promoter activity in lag and stationary phases of growth are known to be different [33].
- The translation rate of the unneeded protein matches that of the average translation rate in the host organism. This assumption is clearly not true under conditions of poor codon adaptability with the host organism [9].
- The fitness cost of generating mRNA transcripts is very small compared to the cost of protein expression.
- The protein translation rate does not depend on growth rate. This assumption fails under conditions of very weak growth [10].
- The fraction of dry cell weight that is protein is invariant with respect to growth rate. In fact, in *E. coli* the protein fraction increases slightly under conditions of weak growth [7], [31] and thus may slightly change the slope of the correlation between growth rate and unnecessary protein fraction at very low growth rates.

## Results

### Ribosome allocation model predicts gene expression cost on growth rates

We chose eGFP expression as an initial test of the ribosome allocation model because it is highly soluble, non-toxic, and activity can be easily assayed. eGFP was expressed using a medium copy number plasmid with a constitutive, insulated promoter [34]. We then created double mutant libraries of this promoter at the −35 and −10 transcriptional start sites and screened colonies for fluorescence intensity visually. We chose a total of 22 clones spanning a range of eGFP expression (**Table S1**) and transformed them into *E. coli* TUNER.

**Figure 1:**
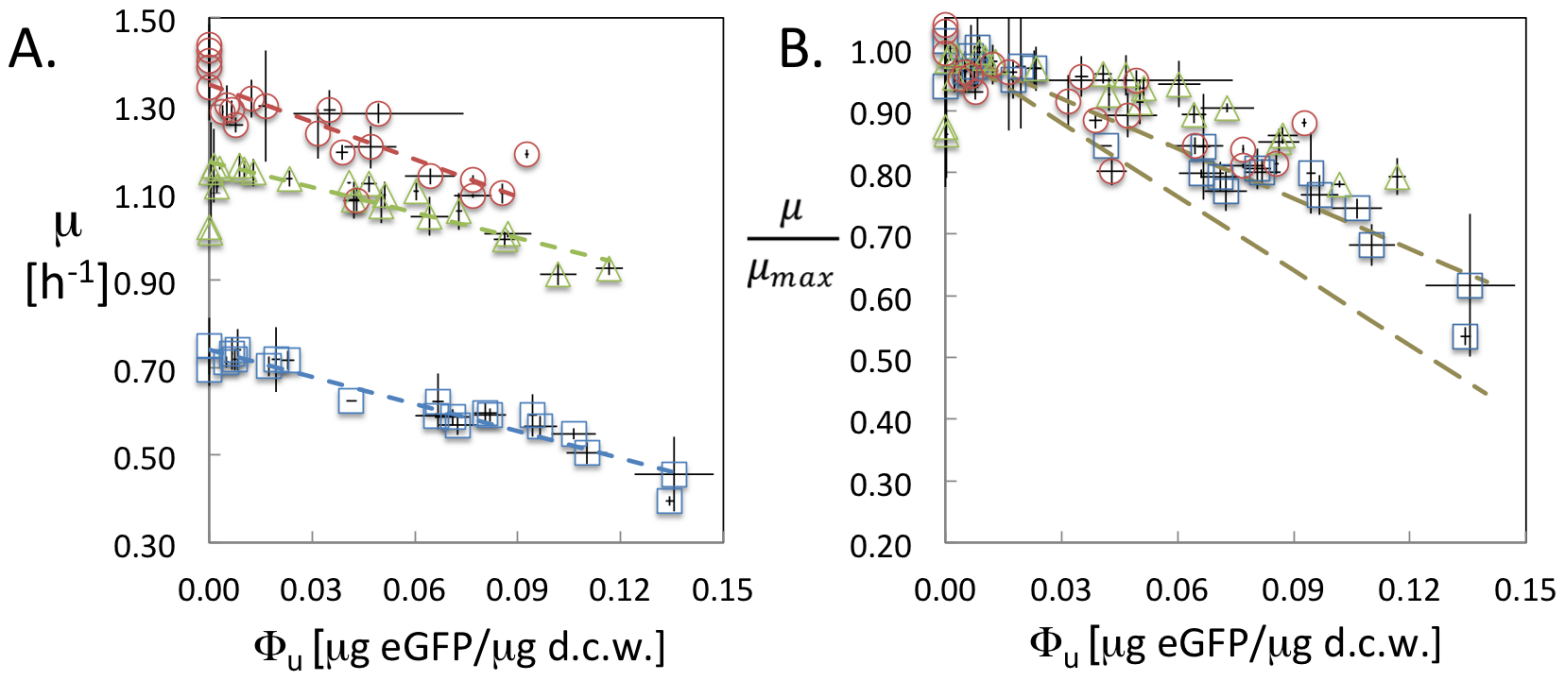
Growth rate as a function of eGFP expression in different growth media. **A**. Growth rate plotted as a function of eGFP mass fraction of dry cell weight (d.c.w.) for promoter variants grown in M9 (blue open squares), M9-CA (green open triangles), and LB (red open circles). Dashed lines are best linear fits of each dataset. **B**. Relative growth rate plotted as a function of eGFP mass fraction of d.c.w. for promoter variants. The growth rate is normalized to the maximum growth rate in the absence of gene expression. The dashed olive lines represent the range of values from theoretical predictions. Error bars represent one standard deviation of at least two independent measurements.

We grew these constructs aerobically at 37°C in media with three different nutritional capacities (M9+Glucose (M9), M9+Glucose+Casamino Acids (M9-CA), and Luria Broth (LB)). For each of these 22 promoter variants we confirmed unimodal eGFP expression as judged by flow cytometry (**Figure S1**). For two representative variants, we confirmed that the plasmid marker expression is insignificant compared to eGFP expression by quantifying mass of TEM-1 BLA relative to total dry cell weight in different growth media (**Table S4**). We then recorded growth rates (μ) and the mass of eGFP relative to total dry cell weight (Φ_U_) during exponential growth of each construct in these different media (**Table S1**). For each media tested there was a clear and striking linear relationship between growth rate and the fraction of protein expressed (**Figure 1A**). The best fits of the unnormalized slopes through each dataset ranged from 2.07 µg DCW per µg eGFP per hr for constructs grown on M9 (R^2^ = 0.93) to 2.85 µg DCW per µg eGFP per hr for constructs grown on LB (R^2^ = 0.70).

When these growth rates were normalized to the maximum growth rate supported by the specific media in the absence of protein expression, the data collapsed onto a single line predicted by ribosome allocation theory (**Figure 1B**):
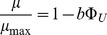
(8)


Where µ_max_ is the growth rate in the absence of protein expression, and *b* is a slope depending solely on two measurable parameters: the protein fraction of the dry weight of a cell, and fraction of the proteome that is not growth-associated. According to previous experimental results [7], [31], both parameters are expected to have weak dependencies on the growth rate that are neglected in this analysis. The best fit through the entire dataset was 2.33 µg DCW per µg eGFP (R^2^ = 0.76), slightly outside the lower bound of the theoretical prediction (range 2.7–4.0 µg DCW per µg eGFP). However, the combined dataset can be fit to the predicted lower bound with only minimal differences in the coefficient of determination (R^2^ = 0.71).

**Figure 2:**
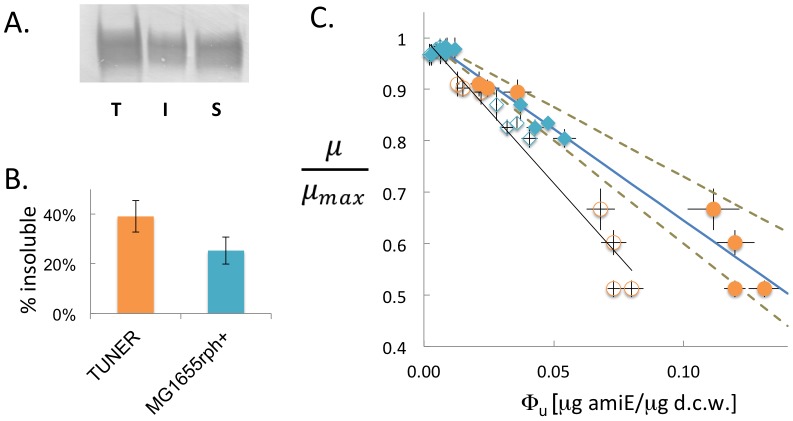
Growth rate dependence on amidase expression in different *E. coli* strains. **A**. Sample Western blot of pJK_proB_amiE expressed in *E. coli* TUNER. T – total cell lysate, I – insoluble fraction of the cell lysate, S – soluble fraction of cell lysate. **B**. Quantification of insoluble amiE relative to total amiE expressed for plasmid pJK_proB_amiE expressed in TUNER and in MG1655rph^+^. The percentage of insoluble amiE was determined by Western blots, while the absolute amount of soluble amiE was determined by enzyme assay of total cell lysates. **C**. Relative growth rate plotted as a function of soluble (open symbols) or total (insoluble plus soluble; closed symbols) amiE mass fraction of d.c.w. for promoter variants grown in M9-CA for *E. coli* TUNER (orange circles) or MG1655rph^+^ (blue diamonds). Error bars represent one standard deviation of at least three independent measurements. The solid lines represent linear best fits through the combined datasets. The dashed olive lines represent the range of values from theoretical predictions.

To test whether this ribosome allocation model holds for another heterologous protein, we measured growth rates and protein expression of an aliphatic amidase amiE from *Pseudomonas aeruginosa*. amiE was chosen because it is thermally stable, has previously been expressed in *E. coli*, and has an activity assay with no background in cell lysates [35]. We chose 9 different promoter variants spanning the range of promoter activities tested in the eGFP samples above, which we cloned in front of *amiE* and transformed into two different strains of *E. coli* (TUNER and MG1655rph^+^). Our quantification of amiE is an activity assay on soluble cell extracts; because of this, any insoluble amiE produced by cells would result in a higher apparent fitness cost to gene expression. To confirm that all of the amidase expressed solubly, we used Western blotting to quantify the fraction of soluble and insoluble amiE from TUNER - expressing the separate plasmids pJK_pro6_amiE and pJK_proB_amiE. In both cases there was a significant insoluble protein fraction (**Figure 2A**). This experiment was repeated in the MG1655rph^+^ strain with similar results: quantification of the insoluble fraction by gel densitometry leads to 39±6% of the total amiE protein is expressed insolubly in TUNER pJK_proB_amiE compared to an insoluble percentage of 25±5% for the MG1655rph^+^ pJK_proB_amiE strain (**Figure 2B**).

Next, we determined growth rates and amidase fraction of total cell weight for each construct at 37°C in M9-CA under aerobic conditions (**Table S2**). In the MG1655rph^+^ strain, all variants were able to support exponential growth. By contrast, expression driven from the two strongest predicted promoter variants, proK1 and proK3, had severe growth defects in the TUNER strain and we were unable to fit exponential growth curves to their growth data (**Figure S2**). When the relative growth rate was plotted against fraction of amidase expressed, the data from the two different strains again collapsed onto a single line consistent with theoretical predictions (**Figure 2A**). Including the insoluble amount of amidase in a balancing of growth rate and protein expression results in a best fit of this slope of 3.55 µg DCW per µg AmiE (R^2^ = 0.97), well within the range predicted by theory (**Figure 2C**). Thus, this scaling law appears to hold even for cases where a significant fraction of the protein is expressed insolubly [36].

### Promoter activity scales with growth rates according to ribosome allocation theory

We then asked to what extent the non-regulated promoter activity correlates across the different media tested in our dataset. From mass balance considerations of protein and mRNA amounts per unit mass, one can derive the following:
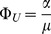
(17)


Where the bundled parameter α, a wholistic measure of promoter activity, is a function of the transcriptional efficiency per gene copy, the gene copy number normalized to cell mass, the translational rate per mRNA transcript, and the mRNA degradation rate ([37]; See Theory). The inverse dependence on growth rate describes the effect of protein dilution by growth under conditions where protein degradation is minimal, a common assumption consistent with other experimental results in bacteria [38]. A null hypothesis is that this bundled promoter activity is independent of growth rate. This hypothesis predicts that, for each unique promoter variant, α would be equivalent for each growth condition and could be determined solely from the protein expression data by:
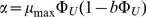
(19)


**Figure 3:**
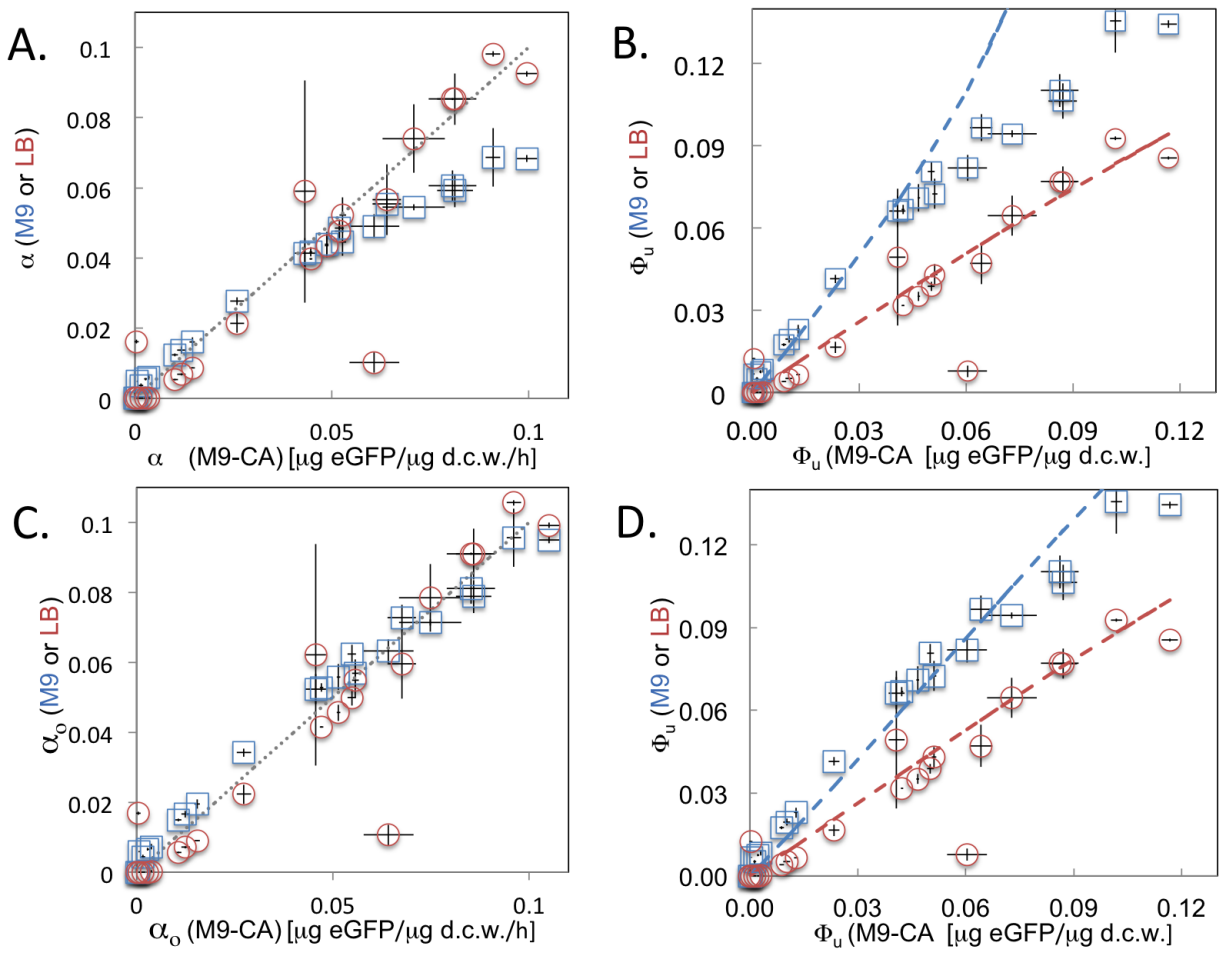
Experimental validation of predicted promoter strengths. For each promoter variant driving eGFP expression, the bundled promoter activity α (**A**), α_o_ (**C**), and eGFP mass fraction (**B**,**D**) of cells grown in M9-CA are plotted against values of cells grown in M9 (blue open squares) or LB (red open circles). Dashed lines indicate the predictions from the promoter activity model in the absence (**A,B**) or presence (**C,D**) of a correction term for promoter activity under weaker growth rates. Note the strong deviations from prediction in the absence of the correction term when cells are grown in M9. Error bars are one standard deviation from at least two independent measurements.

To test this null hypothesis, we compared measured promoter activities from the M9 and LB cases as compared to the M9-CA case (**Figure 3A–B**). This simple model was sufficient to explain the entire range of promoter activities in LB and many of the promoter activities for the M9 dataset as compared to expression in M9-CA. However the model significantly over-predicted promoter activities for the M9 media for promoters with eGFP fractions above 0.07 µg eGFP per µg D.C.W. and growth rates below 0.59 h^−1^. Since activity of the strongest promoters are still well described by the datasets between the LB and M9-CA datasets, we propose that the low growth rates of the strains in M9 media is the cause for deviations from theoretical predictions. Indeed, including a correction term for promoter activity under conditions of weak growth rates, as recently proposed [32], leads to almost perfect agreement between model and experimental results (**Figure 3C–D**). While future work is needed to elucidate the molecular details of the bundled promoter activity under conditions of very high gene expression and low growth rates [10], [39], the promoter activity model presented sufficiently explains eGFP fraction variance across different growth conditions. Accordingly, this model allows for a quantitative prediction of the interrelationship between promoter strength, gene expression, and growth rate for a given genetic construct using two fixed and one single fitted parameter.

### Gene expression cost can significantly constrain pathway flux in metabolic engineering

**Figure 4:**
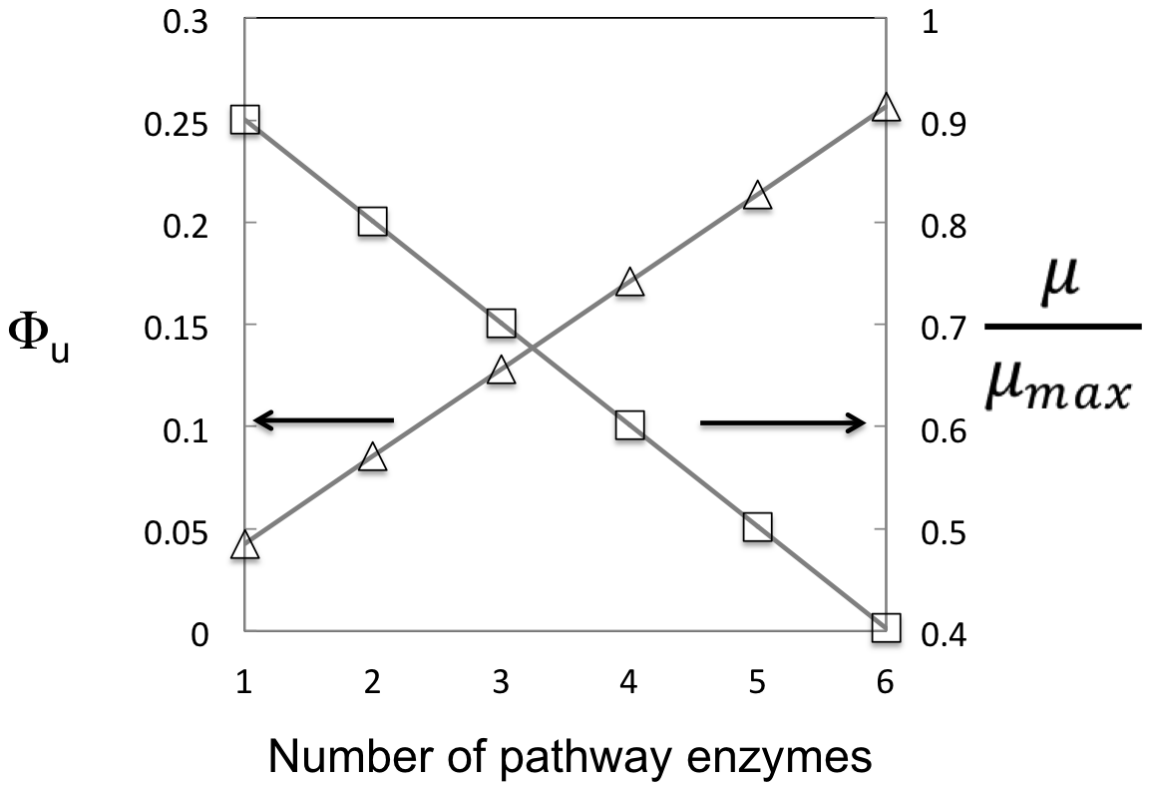
The protein fraction of dry cell weight (Φ_U_, triangles) and relative growth rate (μ/μ_max_, squares) necessary to support a flux of 10 mmol product per gDCW per h is plotted against the number of pathway enzymes involved in a secondary metabolic pathway. Each enzyme is assumed to have a turnover number of 2.6 s^−1^ (the median value for secondary pathway enzymes) and have a molecular weight of 40 kDa. Because saturating kinetics and reaction irreversibility are assumed, this is strictly a lower bound on the fitness cost. Lines are guides for the eye.

Metabolic engineering has increasingly moved from enhancing or rerouting primary metabolism to donating heterologous pathways composed of enzymes from secondary metabolism. It is well known that such enzymes are often less catalytically efficient than enzymes from primary metabolism [40]. Based on the results presented here, we have estimated the lower bound of the fitness cost of expressing a metabolic pathway comprised of enzymes from secondary metabolism (**Figure 4**). For simplicity we assume that each enzyme has a multiple species median value for its turnover rate. Enzymes in a model three-step pathway need to be expressed to at least 12% by weight in order to support a flux of 10 mmol product per g dcw per h, a flux value close to aerobic glycolytic flux in *E. coli*. Expressing five or more heterologous enzymes in a pathway, even if balanced, would result in a growth rate less than 0.5 μ_max_, significantly hampering growth rate and hence volumetric productivities. It is important to note that these calculated values are a strict lower bound of the fitness cost: the analysis does not account for the reversibility of individual reactions or for non-saturating kinetics [41]. Inclusion of these terms would be expected to increase the cost of expressing such a pathway. Historically, one way that metabolic engineers have circumvented this fitness cost is by expressing metabolic pathways on inducible promoters. Thus, the fitness cost of gene expression is not borne until the end of the growth cycle and is accordingly minimized. However, recent drives for continuous fermentations and resultant growth-phase associated product formation places an increasing importance on satisfying these constraints.

## Discussion and Conclusion

In this work we have rigorously quantified the fitness cost to gene expression by systematically varying promoter strengths driving expression of two separate protein systems. We have developed datasets for three different media and for two different *E. coli* strains, and have shown that fitness cost of gene expression is consistent with a theory based on ribosome allocation. One of these proteins, amiE, expressed in both soluble and insoluble form. Thus, this ribosome allocation theory is consistent with experimental results even under conditions where a sizeable fraction of the protein is expressed insolubly. We have developed a simple measure of promoter activity and shown that this can be used to predict amount of expression of a given genetic construct across different growth media. Finally, we have estimated the fitness cost necessary to support high flux through a secondary metabolic pathway. This estimated fitness cost suggests a serious limitation in the ability to support high flux through a pathway comprised of enzymes with weak catalytic efficiencies, such as those from secondary metabolic pathways.

Although we have rigorously assessed the fitness cost of protein expression for synthetic promoters driving expression of two separate genes in *E. coli*, we speculate as to how broadly this fitness scaling law can be applied. The foundation of the integrative model is based on the ideal that a cell is able to allocate its ribosomal resources effectively. The linear relationship between decreasing growth rate and unnecessary protein expression should hold true for any organism that has a linear relationship between RNA:protein ratio and specific growth rate that is the hallmark of efficient ribosome allocation. For example, this correlation holds for many bacteria, yeast (including *S. cerevisiae)*, but not the algae *Prototheca zopfii* [42]. Furthermore, many commonly used knock out strains in metabolic engineering have severe growth defects and/or non-exponential growth phases, or may otherwise not be able to allocate their proteome efficiently in response to changing growth conditions [8]. Thus, we predict that most lab-adapted bacterial strains and several model eukaryotic microorganisms should obey the proposed scaling law.

The slope of the linear relationship in the scaling law (parameter b in equation (8); Theory) is a product of the fraction of dry cell weight that is protein and a term accounting for the growth invariant portion of the proteome and the ribosomal fraction of the proteome under conditions of no growth. These values should be somewhat growth condition (e.g. anaerobic) and species-specific but the slope should not vary considerably beyond 3. For example, the nitrogen percentage of microbial dry cell weight between species ranges between 7.5–14% [43], suggesting a less than 2-fold variation in the protein contribution to dry cell weight. The growth invariant portion of the proteome was found empirically for *E. coli* [7] at slightly more than 50%. While it is unclear how much this percentage will vary among different organisms or different growth conditions, it is tough to imagine a significantly greater fraction among heterotrophs. An additional area where this model can fail is if the net translation rate of the unnecessary protein is much lower than that of the remainder of the proteome, for example if the mRNA encoding the protein has poor codon adaptability. In such a case, as a given ribosome is occupied for a longer time to synthesize the same protein, the model would predict that lower translation rates result in greater fitness cost controlling for protein expression levels.

While more detailed and rigorous model parameterization can alleviate some of the discussed limitations and enhance general utility of the model, nevertheless we have confirmed the model set forth by Scott et al. [7] for two different protein systems in *E. coli*. This relationship appears to set a lower bound on the fitness cost of expressing heterologous protein. Furthermore, we have shown that combining the Scott model with a simple promoter model can be used to quantitatively predict the interrelationship between gene expression, growth, and promoter strength across different media conditions and genes with a single fitted parameter. As such, this work has broad implications for the fields of applied microbial physiology, biochemical engineering, metabolic engineering, and synthetic biology.

## Supporting Information

Figure S1 **Promoter strength of plasmid variants expressing eGFP as judged by flow cytometry.** Samples were grown from an initial OD_600_ = 0.02 in M9-CA at 37°C in deep well plates for 3 h. Then, cells were diluted 50× into PBS and fluorescence immediately measured on a flow cytometer using a 488 nm laser and fluorescence channel FL-1 equipped with a 510±7.5 nm filter. Histograms of five representative clones are shown in order of increasing fluorescence: proK17 – light purple, proK14 – blue, proK11 – light green, pro6 – dark purple, j23150* - orange. All promoter variants supported eGFP expression in unimodal distributions.
(TIFF)

Figure S2 **OD_600_ vs. Time (panel A) and ln(OD_600_) vs. time (panel B) for selected plasmid variants expressing amiE in** ***E. coli*** **TUNER at 37°C in M9-CA.** Sample variants shown are proK17 (green triangles), proK9 (red squares), pro9 (blue diamonds), and proK1 (blue circles). In **panel B**, dashed lines indicate best fits for growth rate determination. proK1 and proK3 (not shown) showed severe growth defects and their growth curves could not be fit to a single exponential growth rate.
(TIFF)

Figure S3 **Quantification of amiE amounts in cell lysates.** (**A.**) AmiE was overexpressed in *E. coli* and purified on a Talon metal affinity resin (Clontech). Denaturing gel electrophoresis shows a single band right below the 40 kDa marker on a PagePlus MW ladder, consistent with the 39 kDa MW of AmiE. (**B.**) The phenol nitroprusside method was used to determine free ammonia liberated by amidase activity on 50 mM acetamide. The colorimetric reaction was monitored at 35°C for 40 min by A_625_ measurements in a Synergy H1 Spectrophotometer until the reaction stabilized – usually in 20 minutes. This dataset shows lysate from *E. coli* MG1655rph^+^ expressing pJK_proB_amiE that had been incubated with 50 mM acetamide for 10 min at rt. (**C.**) A_625_ vs. incubation time of purified amiE at 7 nM (blue open diamonds) and 10 nM (red open circles). Plotting reaction velocities at different enzyme concentrations allows generation of a standard curve. (**D.**) Activity measurements were taken of lysates over 25 minutes. The reaction velocities were used to quantify amiE amounts by comparing to the standard curve. Lysate was diluted to fit within the linear range of the calibration curve. Representative data is shown here of two samples: *E. coli* MG1655rph^+^ pJK_proB_amiE (orange open diamonds) and pJK_proK14_amiE (purple open circles).
(TIFF)

Table S1 **Summary of growth rate and protein expression for** ***E. coli*** **TUNER harboring eGFP plasmid variants.** The reported error is one standard deviation. The proB, pro6, and j23150* promoters sequences have been previously described.
(DOCX)

Table S2 **List of amidase sequences tested, their growth rates, and fraction of** ***soluble*** **amiE expressed per D.C.W.** The reported error is one standard deviation.
(DOCX)

Table S3 **Conversion factors between OD_600_ and dry cell weight as a function of strain and media condition.** Errors are listed as one standard deviation.
(DOCX)

Table S4 **Summary of BLA marker expression for** ***E. coli*** **TUNER harboring selected eGFP plasmid variants in M9 and LB.** The reported error is one standard deviation of two independent experiments.
(DOCX)

Note S1 **DNA and protein-encoding sequences of constructs used for this study.**
(DOCX)
