# Supplementary figures and images for: The Interrelationship between Promoter Strength, Gene Expression, and Growth Rate
Source: PLoS One. 2014 Oct 6;9(10):e109105. doi: 10.1371/journal.pone.0109105 (PMC4186888; doi:10.1371/journal.pone.0109105)

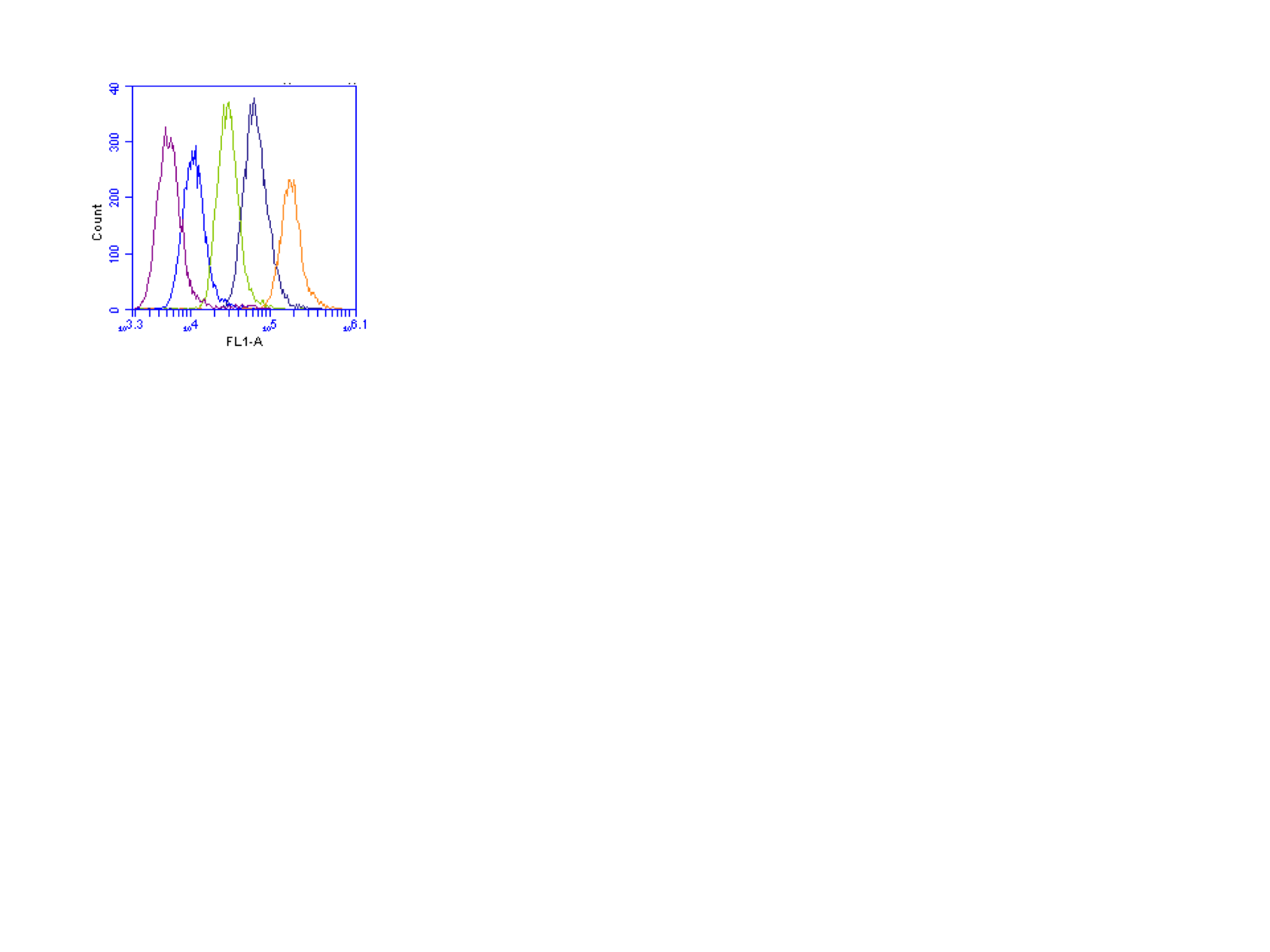

Supplement: Figure S1 — Promoter strength of plasmid variants expressing eGFP as judged by flow cytometry. Samples were grown from an initial OD600 = 0.02 in M9-CA at 37°C in deep well plates for 3 h. Then, cells were diluted 50× into PBS and fluorescence immediately measured on a flow cytometer using a 488 nm laser and fluorescence channel FL-1 equipped with a 510±7.5 nm filter. Histograms of five representative clones are shown in order of increasing fluorescence: proK17 – light purple, proK14 – blue, proK11 – light green, pro6 – dark purple, j23150* - orange. All promoter variants supported eGFP expression in unimodal distributions. (TIFF) [file pone.0109105.s001.tiff]

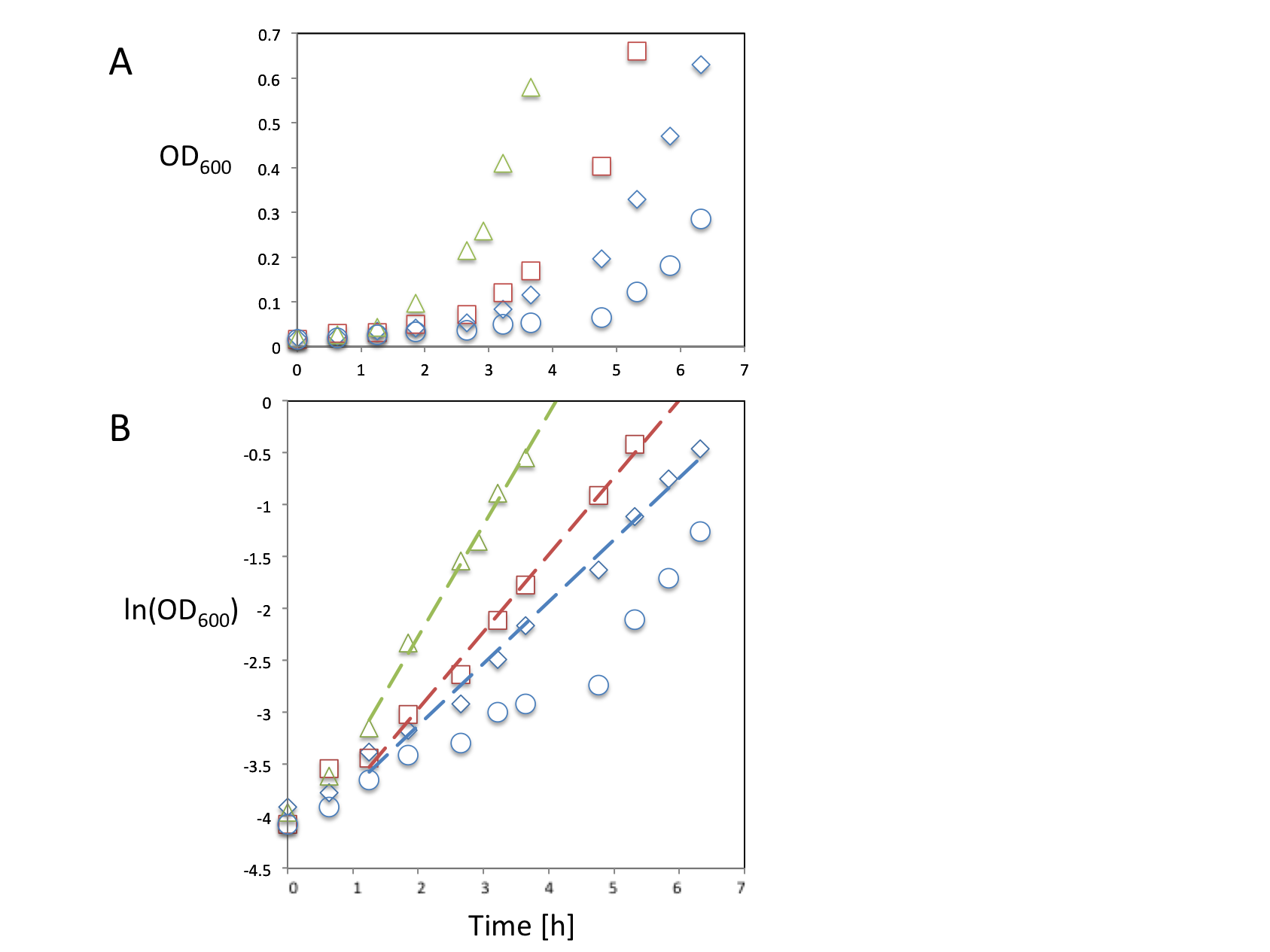

Supplement: Figure S2 — OD600 vs. Time (panel A) and ln(OD600) vs. time (panel B) for selected plasmid variants expressing amiE in E. coli TUNER at 37°C in M9-CA. Sample variants shown are proK17 (green triangles), proK9 (red squares), pro9 (blue diamonds), and proK1 (blue circles). In panel B, dashed lines indicate best fits for growth rate determination. proK1 and proK3 (not shown) showed severe growth defects and their growth curves could not be fit to a single exponential growth rate. (TIFF) [file pone.0109105.s002.tiff]

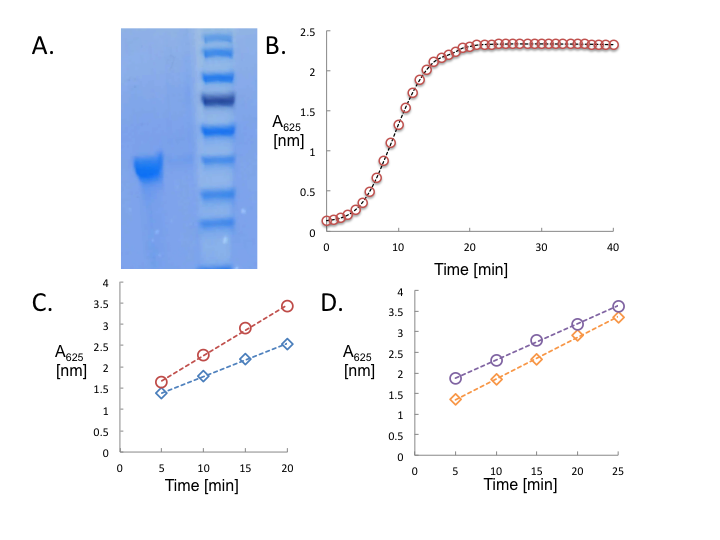

Supplement: Figure S3 — Quantification of amiE amounts in cell lysates. (A.) AmiE was overexpressed in E. coli and purified on a Talon metal affinity resin (Clontech). Denaturing gel electrophoresis shows a single band right below the 40 kDa marker on a PagePlus MW ladder, consistent with the 39 kDa MW of AmiE. (B.) The phenol nitroprusside method was used to determine free ammonia liberated by amidase activity on 50 mM acetamide. The colorimetric reaction was monitored at 35°C for 40 min by A625 measurements in a Synergy H1 Spectrophotometer until the reaction stabilized – usually in 20 minutes. This dataset shows lysate from E. coli MG1655rph+ expressing pJK_proB_amiE that had been incubated with 50 mM acetamide for 10 min at rt. (C.) A625 vs. incubation time of purified amiE at 7 nM (blue open diamonds) and 10 nM (red open circles). Plotting reaction velocities at different enzyme concentrations allows generation of a standard curve. (D.) Activity measurements were taken of lysates over 25 minutes. The reaction velocities were used to quantify amiE amounts by comparing to the standard curve. Lysate was diluted to fit within the linear range of the calibration curve. Representative data is shown here of two samples: E. coli MG1655rph+ pJK_proB_amiE (orange open diamonds) and pJK_proK14_amiE (purple open circles). (TIFF) [file pone.0109105.s003.tiff]
