# Supplementary material for: The Interrelationship between Promoter Strength, Gene Expression, and Growth Rate
Source: PLoS One. 2014 Oct 6;9(10):e109105. doi: 10.1371/journal.pone.0109105 (PMC4186888; doi:10.1371/journal.pone.0109105)
Supplement: Table S3 — Conversion factors between OD600 and dry cell weight as a function of strain and media condition. Errors are listed as one standard deviation. (DOCX) [file pone.0109105.s006.docx]

| *E. coli* Strain | Media | | Conversion Factor [(gDCW/L)-cm/OD_600_] |
| --- | --- | --- | --- |
| TUNER | | M9 | *0.56±0.05* |
| TUNER | | M9-CA | *0.55±0.02* |
| TUNER | | LB | *0.46±0.02* |
| MG1655rph^+^ | | M9-CA | 0.528*±0.004* |
| TUNER pJK_proK6_amiE | | M9-CA | *0.50±0.05* |
| TUNER pJK_proK17_amiE | | M9-CA | *0.50±0.03* |
