# Supplementary material for: The Interrelationship between Promoter Strength, Gene Expression, and Growth Rate
Source: PLoS One. 2014 Oct 6;9(10):e109105. doi: 10.1371/journal.pone.0109105 (PMC4186888; doi:10.1371/journal.pone.0109105)
Supplement: Note S1 — DNA and protein-encoding sequences of constructs used for this study. (DOCX) [file pone.0109105.s008.docx]

DNA sequence of pJK_proB_eGFP, the base plasmid used in this study.

>pJK_proB_eGFP (4,296bp)

ATCCGGATATAGTTCCTCCTTTCAGCAAAAAACCCCTCAAGACCCGTTTAGAGGCCCCAAGGGGTTATGCTAGTTATTGCTCAGCGGTGGCAGCAGCCAACTCAGCTTCCTTTCGGGCTTTGTTAGCAGCCGGATCTCAGTGGTGGTGGTGGTGGTGCTCGAGTTTGTATAGTTCATCCATGCCATGTGTAATCCCAGCAGCTGTTACAAACTCAAGAAGGACCATGTGGTCTCTCTTTTCGTTGGGATCTTTCGAAAGGGCAGATTGTGTGGACAGGTAATGGTTGTCTGGTAAAAGGACAGGGCCATCGCCAATTGGAGTATTTTGTTGATAATGGTCTGCTAGTTGAACGCTTCCATCTTCAATGTTGTGTCTAATTTTGAAGTTAACTTTGATTCCATTCTTTTGTTTGTCTGCCATGATGTATACATTGTGTGAGTTATAGTTGTATTCCAATTTGTGTCCAAGAATGTTTCCATCTTCTTTAAAATCAATACCTTTTAACTCGATTCTATTAACAAGGGTATCACCTTCAAACTTGACTTCAGCACGTGTCTTGTAGTTCCCGTCATCTTTGAAAAATATAGTTCTTTCCTGTACATAACCTTCGGGCATGGCACTCTTGAAAAAGTCATGCTGTTTCATATGATCTGGGTATCTCGCAAAGCATTGAACACCATAACCGAAAGTAGTGACAAGTGTTGGCCATGGAACAGGTAGTTTTCCAGTAGTGCAAATAAATTTAAGGGTAAGTTTTCCGTATGTTGCATCACCTTCACCCTCTCCACTGACAGAAAATTTGTGCCCATTAACATCACCATCTAATTCAACAAGAATTGGGACAACTCCAGTGAAAAGTTCTTCTCCTTTACGCATATGTATATCTCCTTCTTAAAGTTAAACAAAATTATTTCTAGAAAGTTAAACAAAATTATTTGTAGAGGGAAACCGTTGTGGTCTCCCTGAATATATATTACGAGCCTTATGCATGCCCGTAAAGTTATCCAGCAACCACTCATAGACCTAGGGCAGCAGATAGGGACGACGTGGTGTTAGCTGTGGCCGGCATGGCGGCCCCACGGGTGCGCATGATCGTGCTCCTGTCGTTGAGGACCCGGCTAGGCTGGCGGGGTTGCCTTACTGGTTAGCAGAATGAATCACCGATACGCGAGCGAACGTGAAGCGACTGCTGCTGCAAAACGTCTGCGACCTGAGCAACAACATGAATGGTCTTCGGTTTCCGTGTTTCGTAAAGTCTGGAAACGCGGAAGTCAGCGCCCTGCACCATTATGTTCCGGATCTGCATCGCAGGATGCTGCTGGCTACCCTGTGGAACACCTACATCTGTATTAACGAAGCGCTGGCATTGACCCTGAGTGATTTTTCTCTGGTCCCGCCGCATCCATACCGCCAGTTGTTTACCCTCACAACGTTCCAGTAACCGGGCATGTTCATCATCAGTAACCCGTATCGTGAGCATCCTCTCTCGTTTCATCGGTATCATTACCCCCATGAACAGAAATCCCCCTTACACGGAGGCATCAGTGACCAAACAGGAAAAAACCGCCCTTAACATGGCCCGCTTTATCAGAAGCCAGACATTAACGCTTCTGGAGAAACTCAACGAGCTGGACGCGGATGAACAGGCAGACATCTGTGAATCGCTTCACGACCACGCTGATGAGCTTTACCGCAGCTGCCTCGCGCGTTTCGGTGATGACGGTGAAAACCTCTGACACATGCAGCTCCCGGAGACGGTCACAGCTTGTCTGTAAGCGGATGCCGGGAGCAGACAAGCCCGTCAGGGCGCGTCAGCGGGTGTTGGCGGGTGTCGGGGCGCAGCCATGACCCAGTCACGTAGCGATAGCGGAGTGTATACTGGCTTAACTATGCGGCATCAGAGCAGATTGTACTGAGAGTGCACCATATATGCGGTGTGAAATACCGCACAGATGCGTAAGGAGAAAATACCGCATCAGGCGCTCTTCCGCTTCCTCGCTCACTGACTCGCTGCGCTCGGTCGTTCGGCTGCGGCGAGCGGTATCAGCTCACTCAAAGGCGGTAATACGGTTATCCACAGAATCAGGGGATAACGCAGGAAAGAACATGTGAGCAAAAGGCCAGCAAAAGGCCAGGAACCGTAAAAAGGCCGCGTTGCTGGCGTTTTTCCATAGGCTCCGCCCCCCTGACGAGCATCACAAAAATCGACGCTCAAGTCAGAGGTGGCGAAACCCGACAGGACTATAAAGATACCAGGCGTTTCCCCCTGGAAGCTCCCTCGTGCGCTCTCCTGTTCCGACCCTGCCGCTTACCGGATACCTGTCCGCCTTTCTCCCTTCGGGAAGCGTGGCGCTTTCTCATAGCTCACGCTGTAGGTATCTCAGTTCGGTGTAGGTCGTTCGCTCCAAGCTGGGCTGTGTGCACGAACCCCCCGTTCAGCCCGACCGCTGCGCCTTATCCGGTAACTATCGTCTTGAGTCCAACCCGGTAAGACACGACTTATCGCCACTGGCAGCAGCCACTGGTAACAGGATTAGCAGAGCGAGGTATGTAGGCGGTGCTACAGAGTTCTTGAAGTGGTGGCCTAACTACGGCTACACTAGAAGGACAGTATTTGGTATCTGCGCTCTGCTGAAGCCAGTTACCTTCGGAAAAAGAGTTGGTAGCTCTTGATCCGGCAAACAAACCACCGCTGGTAGCGGTGGTTTTTTTGTTTGCAAGCAGCAGATTACGCGCAGAAAAAAAGGATCTCAAGAAGATCCTTTGATCTTTTCTACGGGGTCTGACGCTCAGTGGAACGAAAACTCACGTTAAGGGATTTTGGTCATGAACAATAAAACTGTCTGCTTACATAAACAGTAATACAAGGGGTGTTATGAGTATTCAACATTTCCGTGTCGCCCTTATTCCCTTTTTTGCGGCATTTTGCCTTCCTGTTTTTGCTCACCCAGAAACGCTGGTGAAAGTAAAAGATGCTGAAGATCAGTTGGGTGCACGAGTGGGTTACATCGAACTGGATCTCAACAGCGGTAAGATCCTTGAGAGTTTTCGCCCCGAAGAACGTTTTCCAATGATGAGCACTTTTAAAGTTCTGCTATGTGGCGCGGTATTATCCCGTATTGACGCCGGGCAAGAGCAACTCGGTCGCCGCATACACTATTCTCAGAATGACTTGGTTGAGTACTCACCAGTCACAGAAAAGCATCTTACGGATGGCATGACAGTAAGAGAATTATGCAGTGCTGCCATAACCATGAGTGATAACACTGCGGCCAACTTACTTCTGACAACGATCGGAGGACCGAAGGAGCTAACCGCTTTTTTGCACAACATGGGGGATCATGTAACTCGCCTTGATCGTTGGGAACCGGAGCTGAATGAAGCCATACCAAACGACGAGCGTGACACCACGATGCCTGCAGCAATGGCAACAACGTTGCGCAAACTATTAACTGGCGAACTACTTACTCTAGCTTCCCGGCAACAATTAATAGACTGGATGGAGGCGGATAAAGTTGCAGGACCACTTCTGCGCTCGGCCCTTCCGGCTGGCTGGTTTATTGCTGATAAATCTGGAGCCGGTGAGCGTGGGTCTCGCGGTATCATTGCAGCACTGGGGCCAGATGGTAAGCCCTCCCGTATCGTAGTTATCTACACGACGGGGAGTCAGGCAACTATGGATGAACGAAATAGACAGATCGCTGAGATAGGTGCCTCACTGATTAAGCATTGGTAAGAATTAATTCATGAGCGGATACATATTTGAATGTATTTAGAAAAATAAACAAATAGGGGTTCCGCGCACATTTCCCCGAAAAGTGCCACCTGAAATTGTAAACGTTAATATTTTGTTAAAATTCGCGTTAAATTTTTGTTAAATCAGCTCATTTTTTAACCAATAGGCCGAAATCGGCAAAATCCCTTATAAATCAAAAGAATAGACCGAGATAGGGTTGAGTGTTGTTCCAGTTTGGAACAAGAGTCCACTATTAAAGAACGTGGACTCCAACGTCAAAGGGCGAAAAACCGTCTATCAGGGCGATGGCCCACTACGTGAACCATCACCCTAATCAAGTTTTTTGGGGTCGAGGTGCCGTAAAGCACTAAATCGGAACCCTAAAGGGAGCCCCCGATTTAGAGCTTGACGGGGAAAGCCGGCGAACGTGGCGAGAAAGGAAGGGAAGAAAGCGAAAGGAGCGGGCGCTAGGGCGCTGGCAAGTGTAGCGGTCACGCTGCGCGTAACCACCACACCCGCCGCGCTTAATGCGCCGCTACAGGGCGCGTCCCATTCGCCA

Upstream gene architechture for the pJK_proB_eGFP base plasmid.

Promoter proB: Underlined

-10/-35: Red

Start of transcription: Magenta/Bold

RBS: Blue

Start of translation: Green

>proB_rbs_eGFP

CACAGCTAACACCACGTCGTCCCTATCTGCTGCCCTAGGTCTATGAGTGGTTGCTGGATAACTTTACGGGCATGCATAAGGCTCGTAATATATATTCAGGG**A**GACCACAACGGTTTCCCTCTACAAATAATTTTGTTTAACTTTCTAGAAATAATTTTGTTTAACTTTAAGAAGGAGATATACATATGCGTAAAGGAGAAGAACTTTTCACTGGAGTTGTCCCAATTCTTGTTGAATTAGATGGTGATGTTAATGGGCACAAATTTTCTGTCAGTGGAGAGGGTGAAGGTGATGCAACATACGGAAAACTTACCCTTAAATTTATTTGCACTACTGGAAAACTACCTGTTCCATGGCCAACACTTGTCACTACTTTCGGTTATGGTGTTCAATGCTTTGCGAGATACCCAGATCATATGAAACAGCATGACTTTTTCAAGAGTGCCATGCCCGAAGGTTATGTACAGGAAAGAACTATATTTTTCAAAGATGACGGGAACTACAAGACACGTGCTGAAGTCAAGTTTGAAGGTGATACCCTTGTTAATAGAATCGAGTTAAAAGGTATTGATTTTAAAGAAGATGGAAACATTCTTGGACACAAATTGGAATACAACTATAACTCACACAATGTATACATCATGGCAGACAAACAAAAGAATGGAATCAAAGTTAACTTCAAAATTAGACACAACATTGAAGATGGAAGCGTTCAACTAGCAGACCATTATCAACAAAATACTCCAATTGGCGATGGCCCTGTCCTTTTACCAGACAACCATTACCTGTCCACACAATCTGCCCTTTCGAAAGATCCCAACGAAAAGAGAGACCACATGGTCCTTCTTGAGTTTGTAACAGCTGCTGGGATTACACATGGCATGGATGAACTATACAAACTCGAGCACCACCACCACCACCACTGA

Codon-optimized *amiE* gene sequence with flanking NdeI and XhoI restriction sites.

>*amiE*

**CATATG**AGACATGGCGATATTAGCTCGTCAAATGATACCGTAGGCGTAGCCGTGGTGAATTACAAGATGCCGCGTTTACATACTGCTGCTGAAGTCCTGGATAATGCCCGCAAAATTGCGGAAATGATCGTTGGTATGAAGCAAGGTCTGCCGGGCATGGATCTGGTTGTGTTTCCTGAATATTCTTTACAGGGTATTATGTACGACCCTGCTGAAATGATGGAAACAGCCGTGGCGATTCCAGGCGAAGAAACGGAAATCTTTAGCCGTGCTTGTAGAAAAGCAAATGTTTGGGGTGTGTTCTCCCTGACCGGCGAACGTCATGAAGAACACCCTAGAAAGGCACCATACAACACTCTGGTCTTGATCGATAACAACGGTGAAATCGTACAAAAGTACAGAAAGATCATCCCATGGTGTCCGATTGAAGGCTGGTATCCAGGTGGCCAGACATACGTCTCTGAAGGTCCGAAAGGCATGAAGATCTCATTAATTATCTGCGATGACGGTAATTATCCGGAAATTTGGAGAGATTGTGCCATGAAGGGTGCGGAATTGATCGTTCGCTGCCAAGGCTATATGTACCCTGCTAAAGACCAACAAGTTATGATGGCTAAGGCAATGGCCTGGGCGAATAACTGTTATGTCGCTGTAGCAAACGCTGCAGGTTTTGATGGCGTTTATAGCTACTTCGGTCATAGTGCCATTATCGGTTTTGACGGCCGTACTCTGGGTGAATGCGGCGAAGAAGAAATGGGCATTCAATACGCGCAGTTGTCTCTGTCACAAATCCGCGATGCCCGTGCGAATGACCAAAGTCAGAACCATTTGTTTAAAATCTTGCACAGAGGTTACTCCGGTTTGCAGGCTTCGGGCGATGGCGACCGTGGTCTGGCAGAATGCCCATTTGAATTCTACCGTACCTGGGTTACTGATGCTGAAAAGGCAAGAGAAAACGTGGAACGCCTGACTCGCTCCACAACAGGTGTCGCCCAATGCCCAGTAGGTCGTCTGCCGTATGAAGGC**CTCGAG**

Sequences of translated protein sequences used in this study

>eGFP (27.98 kDa)

MRKGEELFTGVVPILVELDGDVNGHKFSVSGEGEGDATYGKLTLKFICTTGKLPVPWPTLVTTFGYGVQCFARYPDHMKQHDFFKSAMPEGYVQERTIFFKDDGNYKTRAEVKFEGDTLVNRIELKGIDFKEDGNILGHKLEYNYNSHNVYIMADKQKNGIKVNFKIRHNIEDGSVQLADHYQQNTPIGDGPVLLPDNHYLSTQSALSKDPNEKRDHMVLLEFVTAAGITHGMDELYKLEHHHHHH

>amiE (39.0 kDa MW) MRHGDISSSNDTVGVAVVNYKMPRLHTAAEVLDNARKIAEMIVGMKQGLPGMDLVVFPEYSLQGIMYDPAEMMETAVAIPGEETEIFSRACRKANVWGVFSLTGERHEEHPRKAPYNTLVLIDNNGEIVQKYRKIIPWCPIEGWYPGGQTYVSEGPKGMKISLIICDDGNYPEIWRDCAMKGAELIVRCQGYMYPAKDQQVMMAKAMAWANNCYVAVANAAGFDGVYSYFGHSAIIGFDGRTLGECGEEEMGIQYAQLSLSQIRDARANDQSQNHLFKILHRGYSGLQASGDGDRGLAECPFEFYRTWVTDAEKARENVERLTRSTTGVAQCPVGRLPYEGLEHHHHHH
