# Supplementary material for: The Interrelationship between Promoter Strength, Gene Expression, and Growth Rate
Source: PLoS One. 2014 Oct 6;9(10):e109105. doi: 10.1371/journal.pone.0109105 (PMC4186888; doi:10.1371/journal.pone.0109105)
Supplement: Supplementary file 4 [file pone.0109105.s004.docx]

			Growth Rate [h⁻¹]			Φu [ug eGFP/ug DCW]		
**Promoter**	**-35 hexamer**	**-10 hexamer**	**M9**	**M9-CA**	**LB**	**M9**	**M9-CA**	**LB**
**proB**	TTTACG	TAATAT	0.56 ± .02	1.05 ± .02	1.19 ± .04	0.097 ± 0.005	0.064 ± 0.003	0.047 ± 0.008
proK21	TTTACA	TAATAT	0.75 ± .07	1.01 ± .08	1.31 ± .04	0.000	0.000 ± 0.001	0.012 ± 0.000
proK20	TTTACG	TATAAT	0.70 ± .04	1.02 ± .11	1.39 ± .02	0.000	0.000	0.000
proK19	TTTACG	TGATAT	0.71 ± .01	1.16 ± .01	1.44 ± .08	0.005 ± 0.001	0.001 ± 0.000	0.000
proK18	TTTACG	TTATAT	0.73 ± .04	1.15 ± .00	1.42 ± .03	0.007 ± 0.000	0.001 ± 0.000	0.000
proK17	TTCCCG	TAATAT	0.72 ± .02	1.11 ± .03	1.40 ± .08	0.008 ± 0.000	0.002 ± 0.000	0.000
proK16	TTTTCG	TAATAT	0.74 ± .05	1.16 ± .02	1.34 ± .08	0.008 + 0.001	0.003 ± 0.000	0.000
proK14	TGTACG	TAATAT	0.70 ± .02	1.16 ± .03	1.28 ± .01	0.017 ± 0.001	0.009 ± 0.001	0.004 ± 0.000
proK13	TTTAAT	TAATAT	0.72 ± .07	1.15 ± .02	1.30 ± .05	0.019 ± 0.001	0.010 ± 0.01	0.005 ± 0.000
proK12	TTGGCG	TAATAT	0.72 ± .02	1.15 ± .00	1.29 ± .02	0.023 ± 0.002	0.013 ± 0.001	0.007 ± 0.000
proK11	TTTAGT	TAATAT	0.62 ± .00	1.13 ± .04	1.30 ± .13	0.041 ± 0.002	0.023 ± 0.002	0.016 ± 0.002
proK10	TTTACG	TACTAT	0.59 ± .01	1.12 ± .02	1.28 ± .02	0.066 ± 0.006	0.041 ± 0.002	0.049 ± 0.025
proK9	TTTACC	TAATAT	0.62 ± .06	1.08 ± .03	1.24 ± .06	0.067 ± 0.002	0.042 ± 0.000	0.032 ± 0.000
proK8	TTTACG	TAGTAT	0.59 ± .02	1.12 ± .04	1.29 ± .04	0.071 ± 0.005	0.047 ± 0.000	0.035 ± 0.002
proK7	TTTACG	TATCAT	0.57 ± .02	1.10 ± .02	1.08 ± .03	0.072 ± 0.005	0.051 ± 0.001	0.043 ± 0.004
proK6	TTGTCG	TAATAT	0.60 ± .02	1.07 ± .04	1.19 ± .02	0.081 ± 0.003	0.050 ± 0.002	0.039 ± 0.002
pro6	TTTACG	TAAAAT	0.59 ± .02	1.10 ± .04	1.25 ± .02	0.082 ± 0.005	0.060 ± 0.006	0.008 ± 0.002
proK4	TTTACG	TACAAT	0.59 ± .05	1.06 ± .01	1.14 ± .02	0.094 ± 0.001	0.073 ± 0.007	0.065 ± 0.007
proK3	TTTACG	TAGAAT	0.55 ± .01	1.01 ± .02	1.09 ± .00	0.106 ± 0.006	0.087 ± 0.004	0.077 ± 0.005
proK2	TTGCCG	TAATAT	0.50 ± .03	0.99 ± .02	1.13 ± .01	0.110 ± 0.006	0.086 ± 0.005	0.077 ± 0.000
j23150*	TTTACG	TATTAT	0.40 ± .01	0.93 ± .03	1.10 ± .02	0.134 ± 0.001	0.117 ± 0.002	0.086 ± 0.001
proK1	TTTACT	TAATAT	0.46 ± .09	0.91 ± .01	1.19 ± .01	0.136 ± 0.012	0.102 ± 0.002	0.093 ± 0.001
