# Supplementary material for: The Interrelationship between Promoter Strength, Gene Expression, and Growth Rate
Source: PLoS One. 2014 Oct 6;9(10):e109105. doi: 10.1371/journal.pone.0109105 (PMC4186888; doi:10.1371/journal.pone.0109105)
Supplement: Supplementary file 5 [file pone.0109105.s005.docx]

Promoter	-35 hexamer	-10 hexamer	Growth Rate [h^{-1}] TUNER	Growth Rate [h^{-1}] MG1655rph^{+}	Φu [μg AmiE/μg DCW] TUNER	Φu [μg AmiE/μg DCW] MG1655rph^{+}
**proB**	TTTACG	TAATAT	0.63 ± 0.02	0.90 ± 0.02	0.073 ± 0.002	0.032 ± 0.002
proK17	TTCCCG	TAATAT	1.12 ± 0.03	1.05 ± 0.02	0.013 ± 0.001	0.002 ± 0.001
proK16	TTTTCG	TAATAT	1.11 ± 0.02	1.07 ± 0.01	0.015 ± 0.003	0.005 ± 0.001
proK14	TGTACG	TAATAT	1.10 ± 0.03	1.07 ± 0.02	0.022 ± 0.003	0.006 ± 0.003
proK9	TTTACC	TAATAT	0.82 ± 0.05	0.91 ± 0.01	0.068 ± 0.006	0.036 ± 0.000
proK6	TTGTCG	TAATAT	0.74 ± 0.03	0.88 ± 0.02	0.073 ± 0.005	0.041 ± 0.003
pro6	TTTACG	TAAAAT	0.63 ± 0.02	0.95 ± 0.03	0.080 ± 0.005	0.028 ± 0.000
proK3	TTTACG	TAGAAT	--	1.07 ± 0.02	--	0.009 ± 0.001
proK1	TTTACT	TAATAT	--	1.06 ± 0.02	--	0.006 ± 0.002
