# Supplementary material for: The Interrelationship between Promoter Strength, Gene Expression, and Growth Rate
Source: PLoS One. 2014 Oct 6;9(10):e109105. doi: 10.1371/journal.pone.0109105 (PMC4186888; doi:10.1371/journal.pone.0109105)
Supplement: Supplementary file 6 [file pone.0109105.s007.docx]

Promoter	-35 hexamer	-10 hexamer	Φu [ug BLA/ug DCW] M9	Φu [ug BLA/ug DCW] LB	Φu [ug eGFP/ug DCW] M9	Φu [ug eGFP/ug DCW] LB
**proB**	TTTACG	TAATAT	.000016 ± .000002	.000060 ± .000012	0.097 ± 0.005	0.047 ± 0.008
proK17	TTCCCG	TAATAT	.000056 ± .000008	.000060 ± .000006	0.008 ± 0.000	0.000
